# Childhood Guillain–Barre syndrome in the SARS‐CoV‐2 era: Is there any causative relation?

**DOI:** 10.1002/ccr3.6772

**Published:** 2022-12-20

**Authors:** Elham Pourbakhtyaran, Morteza Heidari, Masood Ghahvechi Akbari, Mahmoud Mohammadi, Reza Shervin Badv, Gholamreza Zamani, Ali Reza Tavasoli, Zahra Rezaei, Setareh Mamishi, Elmira Haji Esmaeil Memar, Seyyed Mohammad Mahdi Hosseiny, Homa Ghabeli, Roya Haghighi, Mahmoud Reza Ashrafi

**Affiliations:** ^1^ Department of Pediatric Neurology, Pediatrics Center of Excellence, Children's Medical Center Tehran University of Medical Sciences Tehran Iran; ^2^ Myelin Disorders Clinic, Pediatric Neurology Division, Children's Medical Center, Pediatrics Center of Excellence Tehran University of Medical Sciences Tehran Iran; ^3^ Department of Physical Medicine and Rehabilitation, Pediatrics Center of Excellence, Children's Medical Center Tehran University of Medical Sciences Tehran Iran; ^4^ Pediatric Infectious Disease Research Center Tehran University of Medical Sciences Tehran Iran; ^5^ Pediatrics Center of Excellence, Children's Medical Center Tehran University of Medical Sciences Tehran Iran; ^6^ Department of Pediatric Neurology, School of Medicine Hamadan University of medical sciences Hamadan Iran

**Keywords:** Guillain–Barre syndrome, pediatric, SARS‐CoV‐2

## Abstract

We reported an association between SARS‐CoV‐2 infection and Guillain–Barre syndrome (GBS). From 37 patients with GBS, previous SARS‐CoV‐2 clinical clues, including fever, cough, and diarrhea, were recorded in 18 patients. Among them, SARS‐CoV‐2 IgG was detected in seven patients, considered confirmed as cases. SARS‐CoV‐2 PCR was positive in just one patient. Although we found no increase in patient recruitment during the pandemic compared to previous years, our study indicated that SARS‐CoV‐2 is associated with poorer outcomes regarding GBS disability scale and hospital stay.

## Abstract 2

Although the rate of Guillain–Barre syndrome in children associated with SARS‐CoV‐2 was the same as previous years, it would be related to more hospital stay and serious complications that is necessary for pediatric neurologists to mention.

## 1 INTRODUCTION

According to previous reports of neurologic manifestations of coronaviruses, it was expected that we would find these neurological disorders in the severe acute respiratory syndrome coronavirus 2 (SARS‐CoV‐2), including headache, seizures, cerebrovascular disorders, and Guillain–Barre syndrome (GBS).^1^, ^2^

Guillain–Barre syndrome is an immune‐mediated peripheral neuropathy, typically characterized by rapidly progressive bilateral and often symmetrical loss of sensory and motor functions of the limbs. It might also involve respiratory or cranial nerve‐innervated muscles.^3^ GBS is usually preceded by an infection that often has been caused by *Campylobacter jejuni* or other bacterial or viral agents.^4^ GBS has been associated with respiratory viral pathogens such as human coronaviruses^5^; therefore, an outbreak of some viral infections may lead to increased admission of GBS patients.

Based on clinical and electrophysiologic studies, GBS is divided into several subtypes, including acute inflammatory demyelinating polyradiculoneuropathy (AIDP), two axonal forms of GBS including acute motor‐sensory axonal neuropathy (AMSAN), and acute motor axonal neuropathy (AMAN) and Miller Fisher syndrome (MFS).^6^ Preceding infections may result in special clinical and electrophysiological subtypes of GBS.^7^

There are some neurologic complications in children, associated with the severe acute respiratory syndrome coronavirus 2 (SARS‐CoV‐2). A 6‐year‐old male patient had been reported with a rapid progression axonal GBS and positive SARS‐CoV‐2 infection.^8^ A 9‐year‐old boy with reported with positive SARS‐CoV‐2 infection and self‐recovered Guillain–Barré syndrome without immunotherapy.^9^

During the SARS‐CoV‐2 pandemic, we assumed that the incidence of GBS had increased, and the subtypes may change; therefore, we set up a prospective study for neurological manifestations of SARS‐CoV‐2 infections in the Children's Medical Center (CMC), Tehran, which is one of the largest referral pediatric centers in Iran. We reported the clinical finding, electrophysiological subtype, and disease course of all GBS patients during the 17‐month period of study.

## 2 MATERIALS AND METHODS

This prospective study was performed in Children Medical Center, Tehran, Iran, from March 2020 to August 2021. All patients' parents had assigned the consent form. This study was approved by the Ethics Committee of Tehran University of Medical Sciences, (Ethic code: IR.TUMS.VCR.REC. 1399.326).

All admitted children that fulfill the diagnostic criteria for GBS (National Institute of Neurological Disorders and Stroke) enrolled during the study period.^10^ Exclusion criteria for this study were as follows: other neurological diseases, which could affect brain function, sequelae neurologic deficits that could affect the evaluation of GBS severity.

Demographic data, associated symptoms, GBS severity according to disability scale^1^, ^11^ on admission and discharge time, clinical and electrophysiologic features, SARS‐CoV‐2 status, and other important data were recorded in a structured checklist. Disability Scale in GBS is defined as the following: 0. Healthy; 1. Minor signs or symptoms of neuropathy but capable of manual work; 2. Able to walk without the support of a stick but incapable of manual work; 3. Able to walk with a stick, appliance, or support; 4. Confined to bed or chairbound; and 5. Requiring assisted ventilation. 6 Death.^11^

All patients with GBS underwent standard treatment by IVIG as indicated. Data analyzed with suitable statistical methods.

Clinical suspicion of SARS‐CoV‐2 infection, laboratory, radiological, and serological evidence recorded for all patients. Reverse‐transcription polymerase chain reaction (RT‐PCR) of nasopharyngeal swab specimen and serum antibodies (IgM and IgG) was requested for all patients. Patients with positive SARS‐CoV‐2 PCR or antibodies defined as confirmed SARS‐CoV‐2 cases and those with clinical symptoms including fever, diarrhea, and upper respiratory complaints or imaging finding in favor of SARS‐CoV‐2 or contact with confirmed SARS‐CoV‐2 cases before neurologic signs considered as probable SARS‐CoV‐2 cases.^12^

## 3 RESULTS

Of 37 patients with GBS, 28 were male and 9 were female. The mean age was 8.19 ± 3.49 years, ranging from 2.5 to 15 years. The frequency of previous SARS‐CoV‐2 clinical clues, including fever, cough, and diarrhea, was 48%. SARS‐CoV‐2 PCR or antibodies were detected in 7 patients (19%), considered as confirmed cases. Among them, SARS‐CoV‐2 PCR was positive in just 1 patient. The overall frequency of confirmed plus probable SARS‐CoV‐2 in GBS patients was 60% (*N* = 22). The median time from the onset of infection to neurological symptoms was 11.25 ± 8.44 days (ranging from 2 to 30 days). Demographic data and clinical findings according to SARS‐Cov‐2 infection status are noted in Table 1.

**TABLE 1:** Frequency of demographic and clinical findings according to SARS‐Cov‐2 infection status.

	SARS‐CoV‐2 negative (*N* = 15)	Confirmed SARS‐CoV‐2 (*N* = 7)	Confirmed and Probable SARS‐CoV‐2 (*N* = 22)	Total (*N* = 37)
Sex				
Male	12 (80%)	5 (71%)	16 (72)	28 (76%)
Female	3 (20%)	2 (29%)	6 (28%)	9 (24%)
Previous suspected symptoms^a^				
Fever	0	2 (28%)	8 (36%)	8 (22%)
Diarrhea	0	1 (14%)	6 (27%)	6 (16%)
URI	0	2 (28%)	10 (45%)	10 (27%)
Disability scale on admission				
0	0	0	0	0
1	0	0	0	0
2	5 (33%)	0	6 (27%)	11 (30%)
3	7 (47%)	3 (43%)	9 (41%)	16 (43%)
4	3 (20%)	3 (43%)	5 (23%)	8 (22%)
5	0	1 (14%)	2 (9%)	2 (5%)
6	0	0	0	0
Electrodiagnostic findings				
AIDP	4 (26%)	2 (29%)	8 (36%)	12 (32%)
AMAN	10 (67%)	5 (71%)	14 (64%)	24 (65%)
AMSAN	1 (17%)	0	0	1 (3%)
Disability scale at discharge				
0	0	0	0	0
1	0	0	1 (4%)	1 (3%)
2	5 (33%)	1 (14%)	6 (27%)	11 (30%)
3	7 (47%)	4 (57%)	13 (60%)	20 (54%)
4	3 (20%)	2 (29%)	2 (9%)	5 (13%)
5	0	0	0	0
6	0	0	0	0

*Note*: Two confirmed patients had no symptoms.

^a^ Please notice that the number of symptoms does not refer to number of patients, necessarily, as one patient may have more than one symptom.

All patients underwent electrophysiological examination, and acute axonal type motor polyneuropathy was the most prevalent type in both SARS‐CoV‐2‐positive and SARS‐CoV‐2‐negative patients. The second frequent type was acute segmental demyelinating motor polyradiculoneuropathy. The disability scale in most of the patients on admission and on discharge was 3 in 43.2% and 54.1%, respectively.

Acute motor axonal neuropathy was the most frequent subtype of GBS in all our patients (65%), apart from SARS‐CoV‐2 infection status. The second frequent subtype was AIDP (32%), and the AMSAN subtype was detected in just one SARS‐CoV‐2 infection‐negative patient. These findings were not significantly different between SARS‐CoV‐2 infection positive and negative cases (*p* value: 0.42).

Guillain‐Barré syndrome disability scale 4 ≤ out of 6 at presentation in confirmed patients with SARS‐CoV‐2 infection was 57%, in comparison with 20% in SARS‐CoV‐2 infection‐negative patients, which was statistically significant (*p* value: 0.02). One patient was an obese 8‐year‐old boy with a history of fever 14 days before weakness onset and SARS‐CoV‐2 IgG was positive. GBS disability scale (DS) on admission was 5 out of 6. During 24 h of lower limb weakness, the ascending process involved the respiratory system and he was intubated for about 2 weeks and then underwent tracheostomy for 2 months later. Another patient was a 15‐year‐old girl with a history of lupus erythematous and upper respiratory infection symptoms around 3 weeks before weakness; however, SARS‐CoV‐2 antibodies or PCR were negative. She had ascending weakness and diplopia, after 1 week of the disease, she underwent intubation for 3 weeks and then tracheostomy for 1.5 months later. Both patients had refractory hypertension and tachycardia. Treatment was challenging, and they received IVIG. After 4 weeks of treatment, they received high dose of methylprednisolone, due to poor response to IVIG. Electrophysiology study was in favor of acute motor axonal neuropathy in both of them. They had the longest hospital admission time which was around 90 and 60 days, respectively.

The mean of hospital admission time, among confirmed SARS‐CoV‐2 infection patients, was 19.7 days, and among confirmed plus probable SARS‐CoV‐2 infection was 11.8 days. These amounts are significantly higher than 5.5 days in SARS‐CoV‐2 infection‐negative patients (*p* value < 0.05). Hospital stay was longer in around 8‐year‐old patients, with a mean of 20 days. Acute axonal type motor polyneuropathy was associated with the longest admission time (mean 11.78 ± 20.32 days, ranging from 4 to 89 days). This variable was longer in SARS‐CoV‐2 positive in comparison with those with negative SARS‐CoV‐2.

## 4 DISCUSSION

In a study of 81 patients, most patients were diagnosed with AIDP. Three of the patients died at the end of the first month. Three patients had recurrent GBS. Seventy‐four patients received IVIG while eight patients were treated with plasma exchange after IVIG because of ineffective treatment. Seven of the patients were followed without treatment.^4^

Among 49 children with GBS, rapid progression to maximum paralysis was seen in the male gender, while the older age group in pediatrics is expected to endure residual paralysis at 60 days after disease onset. Patients in colder seasons were more likely to have residual paralysis too, compared to warmer seasons.^5^

Among 30 children with GBS diagnosis, in Iran, 12 participants were diagnosed with acute inflammatory demyelinating polyradiculoneuropathy and 18 patients were diagnosed with acute motor axonal neuropathy.^6^ GBS is usually preceded by an infectious viral or bacterial process.^4^ Here, we investigated the association between SARS‐CoV‐2 and GBS in children. Previously, we performed a study from March 2014 to 2017, in a tertiary children's hospital that enrolled 69 children with GBS. The prevalence of GBS in the new study, during the SARS‐CoV‐2 pandemic, is higher; however, this difference is not statistically valuable.

The male to female ratio in our study was 3.1, and among confirmed cases, this ratio was 2.5. in a study by M. Ashrafi et al.^13^ in 2008, in Iran, this ratio was 1.05, which may reflect the higher male involvement during SARS‐CoV‐2 pandemic.

The frequency (60%) of a preceding SARS‐CoV‐2 infection track(confirmed plus probable cases) in our study population was higher than estimates in a multicenter study by Luijten LWG, et al.^14^ on adults with GBS, which was 22%. This may reflect the higher incidence of SARS‐CoV‐2 infection in Iran, during that time, which was about 1 million in 80 million population according to an official report of the ministry of health and medical education of Iran.

Disability scale 4 ≤ of 6 and at presentation in confirmed patients with SARS‐CoV‐2 infection was 57%, in comparison with 20% in SARS‐CoV‐2 infection‐negative patients, which was statistically significant. Just as, we had one intubated patient in confirmed and one in probable cases. These findings along with the longest hospital admission time in SARS‐CoV‐2 infection patients may reflect a poorer prognosis among SARS‐CoV‐2 infection patients.

Acute motor axonal neuropathy was the most frequent subtype of GBS in all our patients (65%), apart from SARS‐CoV‐2 infection status. This finding, is concordant with a study of M. Ashrafi et al.^15^ in Iran and different from studies of other countries, with AIDP predominance.^16^ The second frequent subtype was AIDP (32%) and AMSAN was detected in just one SARS‐CoV‐2 infection‐negative patient. These findings were not significantly different between SARS‐CoV‐2 infection positive and negative cases.

## 5 CONCLUSION

Consistent with previous studies, we found no increase in GBS patient recruitment during the pandemic compared to previous years; however, our study indicated that SARS‐CoV‐2 is associated with poorer outcomes. Since our study was performed with 37 patients, we could not determine the causative relationship. Therefore, we recommend studies with a larger sample size to determine whether there is a causative association or not.

## AUTHOR CONTRIBUTIONS

**M.A., M.H.:** Conceptualization, Methodology, Software. **E.P., M.H., A.R.T.**: Data curation, Writing‐ Original draft preparation. **RSB, M.M, G.R.Z., M.G.A., E.H.E.M., R.H.**: Visualization, Investigation. **M.A., S.M., M.H.:** Supervision. **S.M.M.H., H.G., Z.R.**: Software, Validation. **E.P., M.H.:** Writing‐ Reviewing and Editing. All authors read and approved the final manuscript.

## CONFLICT OF INTEREST

The authors declare that they have no conflict of interest.

## ETHICAL APPROVAL

Ethics approval obtained from the Tehran University of Medical Sciences (approval number: IR.TUMS.VCR.REC.1399.326). Written informed consent was obtained from the patients' parents for inclusion in the study, in accordance with the ethical guidelines by the ethics committee of Tehran University of Medical Sciences.

## CONSENT

Written informed consent was obtained from the patients' parents to publish this report in accordance with the journal's patient consent policy.

## Data Availability

The datasets used and analyzed during the current study are available from the corresponding author on reasonable request.
